# Integration of cuproptosis-related gene signatures in stomach adenocarcinoma: implications for prognostic prediction and therapeutic strategies in cancer drug resistance

**DOI:** 10.1007/s12672-025-02740-8

**Published:** 2025-05-23

**Authors:** Xin Zuo, Youchun Lei, Shan Ou, Xiu Yuan, Peng Shi, Qian Li, Yun Xu

**Affiliations:** Department of Gastroenterology, The Sixth People’s Hospital of Chongqing, Chongqing, 400060 China

**Keywords:** Stomach adenocarcinoma, Cuproptosis, *FDX1*, Prognostic model, Gene signature

## Abstract

**Background:**

Stomach adenocarcinoma (STAD) is a prevalent and aggressive cancer, often diagnosed at later stages, which poses challenges for effective treatment. Despite advancements in cancer therapies, the phenomenon of tumor drug resistance remains a critical hurdle. Recent studies have highlighted cuproptosis, a copper-dependent regulated cell death process, as a potential mechanism in various cancers, including STAD. This study integrates cuproptosis-related gene signatures with clinical features to better predict prognosis and explore potential therapeutic targets, focusing on the role of cuproptosis in overcoming tumor resistance mechanisms.

**Methods:**

Using comprehensive datasets from TCGA-STAD (n = 375 tumor samples, 32 normal samples), GTEx (n = 211 normal gastric tissues), and GEO (GSE84437 and GSE29272), we analyzed the expression of genes associated with cuproptosis. We examined genetic alterations, immune infiltration, and constructed multivariate Cox regression models with clinicopathological covariates (age, gender, TNM stage, histological grade, residual tumor status) to assess the relationship between cuproptosis gene expression and patient survival outcomes, including overall survival (OS), disease-specific survival (DSS), and progression-free interval (PFI). Drug sensitivity analysis was performed using the Genomics of Drug Sensitivity in Cancer (GDSC) database.

**Results:**

Our analysis identified significant upregulation of several cuproptosis-related genes, including *FDX1*, which was correlated with improved prognosis and immune cell infiltration patterns. High expression of *FDX1* was associated with better OS and DSS outcomes. Further genetic alterations, notably in *CDKN2A*, were frequent and linked to poor prognosis, highlighting the complexity of tumor drug resistance in STAD. Prognostic models incorporating *FDX1*, *PDHA1*, and *LIAS* expression stratified patients into distinct risk categories, emphasizing their potential as biomarkers for personalized therapeutic strategies.

**Conclusions:**

This study underscores the importance of cuproptosis-related genes, particularly *FDX1*, in the prognosis and therapeutic response of STAD. By integrating molecular features with clinical data, we offer insights into the potential for overcoming drug resistance in cancer therapy. These findings lay the groundwork for future research into targeted treatments that modulate cuproptosis, offering a novel approach to tackling tumor progression and resistance in STAD.

## Introduction

Stomach adenocarcinoma (STAD) is among the most prevalent and aggressive malignancies globally, being one of the main causes of morbidity and death from cancer [1]. The outlook for STAD is still bleak despite improvements in diagnostic and treatment techniques, largely due to late-stage diagnosis [2], heterogeneity of the disease [3], and limited understanding of its molecular mechanisms [4]. This highlights the urgent need to identify reliable biomarkers and develop robust prognostic models to stratify patients and guide personalized treatment strategies [5].

The crucial role that controlled cell death cascades play in carcinogenesis and cancer development has been highlighted by recent research [6, 7]. Among these, cuproptosis—a novel, copper-dependent mechanism of cell death—has garnered significant attention [8]. Unlike apoptosis and ferroptosis, cuproptosis is induced by copper ion accumulation and directly targets mitochondrial metabolism, making it a unique and promising area of research [9]. Emerging evidence suggests that dysregulation of cuproptosis-related genes is implicated in cancer development and progression, including their role in modulating the tumor microenvironment [10] and influencing therapeutic responses [11, 12]. The interplay between cuproptosis and other cell death mechanisms such as ferroptosis and apoptosis has been shown to be critical in determining cancer cell fate and drug resistance [13]. Nevertheless, the prognostic significance of cuproptosis-related genes in STAD remains poorly understood.

In recent years, advances in high-throughput sequencing and bioinformatics have facilitated the integration of molecular and clinical data, enabling the development of predictive models for cancer prognosis [14]. Several studies have successfully utilized gene expression profiles to identify prognostic signatures in various cancers, yet the integration of cuproptosis-related gene signatures in STAD is still in its infancy [15–17]. For example, prior research has identified alterations in mitochondrial-related genes as significant prognostic markers [18], while other investigations have highlighted immune infiltration role [19] and genomic instability [20] in shaping cancer outcomes. These results underscore the potential of combining molecular signatures with clinical features to improve risk stratification and prognostic accuracy.

This study builds upon these advances by investigating the prognostic value of cuproptosis-related genes in STAD, with a particular focus on their potential role in modulating drug resistance. We hypothesized that cuproptosis-related gene signatures could provide additional prognostic information beyond conventional clinical factors and potentially identify novel therapeutic targets. Using comprehensive datasets from TCGA-STAD, GTEx, and GEO, we examined the expression patterns and genetic alterations of cuproptosis-related genes. We further constructed multivariate prognostic models to predict OS value (overall survival), PFI (progression-free interval) and DSS (disease-specific survival). By integrating cuproptosis-related gene signatures with clinical variables, our investigation aims to provide novel insights into prognostic landscape of STAD and contribute to development of personalized therapeutic approaches.

## Methods

### Data acquisition and preprocessing

STAD gene expression profiles were obtained from The Cancer Genome Atlas (TCGA) database (https://portal.gdc.cancer.gov/), comprising 375 tumor samples and 32 normal tissue samples [21]. We focused on cuproptosis-related genes (DLD, FDX1, LIPT1, DLAT, LIAS, PDHB, MTF1, GLS, CDKN2A, PDHA1) based on recent literature identifying their roles in copper-dependent cell death [22]. Sample inclusion criteria were: primary STAD with available RNA-seq data, complete clinical information, and follow-up data. Samples with missing survival data were excluded.

To enhance our analysis, normal tissue expression data (n = 211 gastric tissue samples) were incorporated from the Genotype-Tissue Expression (GTEx) database [23]. Additional independent validation was conducted using datasets from Gene Expression Omnibus (GEO), specifically GSE84437 using the GPL570 platform and GSE29272 using the GPL96 platform [24]. The datasets were normalized, with RNA-seq data (TCGA and GTEx) transformed to log2(TPM + 1) for consistency. Batch effects between TCGA and GTEx were corrected using the ComBat algorithm from the 'sva' R package. GEO datasets were normalized using the Robust Multi-array Average (RMA) method. Clinical annotations, including OS (overall survival), PFI (progression-free interval) and DSS (disease-specific survival), were haul out for patients with available clinical follow-up data.

The Level 4 gene-level Copy Number Variation (CNV) dataset were also downloaded for all TCGA samples processed using the GISTIC software [25] from the portal GDC (https://portal.gdc.cancer.gov/).

### Analysis of differential gene expression

Differential expression analysis was performed to compare cuproptosis-related gene expression between normal and tumor tissues. Significant differential expression was defined as |log2FoldChange|> 1 and adjusted p-value < 0.05, calculated using the 'limma' R package. Expression differences were visualized using box plots for cuproptosis-related genes across TCGA-STAD and combined TCGA-GTEx datasets. Paired sample comparisons were conducted for TCGA-STAD samples to assess intra-individual expression changes. The Wilcoxon rank-sum test and the Wilcoxon signed-rank test were used to establish statistical significance. To verify expression patterns observed in TCGA-STAD, we analyzed independent GEO datasets with platforms GPL570 and GPL96.

Correlation matrices of gene expression across TCGA-STAD samples were visualized via heatmaps to explore potential co-regulation among cuproptosis-related genes.

### Genetic alteration profiling

The mutation landscape of cuproptosis-related genes was characterized using TCGA-STAD and cBioPortal data, focusing on samples with detectable genetic alterations [26]. Oncoplots were generated to illustrate mutation types and frequencies. Copy number alterations, mutations, and structural variants were summarized across multiple studies to understand the genetic diversity within these genes.

### Prognostic analysis

The predictive relevance of clinical characteristics and genes linked to cuproptosis was assessed using univariate and multivariate Cox proportional hazards models. For multivariate models, we included the following covariates: age, gender, TNM stage, histological grade, and residual tumor status to adjust for potential confounding factors. Reports of 95% CIs and hazard ratios (HRs) were provided for important predictors. Using data from TCGA and the Kaplan–Meier plotter website (n = 876 gastric cancer samples) as an independent validation cohort [27], Kaplan–Meier survival curves displayed the overall survival of STAD individuals stratified by *FDX1* median expression. To test the robustness of our findings, we performed stratified analyses by stage and histological subtype.

### *FDX1* expression and clinicopathological correlations

The association of *FDX1* expression with demographic, clinical, and histopathological parameters was assessed.

### Diagnostic analysis

The diagnostic value of *FDX1* expression was assessed employing ROC curve analysis, with AUC values indicating its capacity to discriminate between tumor and normal tissues.

### Tumor microenvironment and immune infiltration

Immune infiltration was quantified employing CIBERSORT algorithm [28] and ESTIMATE algorithm for various immune cell types in tumors with high or low *FDX1* expression. Correlation analyses were conducted using Spearman's rank correlation to elucidate the association among *FDX1* expression and immune infiltration. The association of *FDX1* with TMB (tumor mutation burden), neoantigen load, and MSI (microsatellite instability) was further explored with Spearman's rank correlation analyses.

### *FDX1* expression and genomic alterations

The difference of *FDX1* expression between genomic alteration categorys (neutral, gain, and loss) was assessed using ANOVA (one-way analysis of variance). The mutation profiles of the 15 most frequently mutated genes in stomach adenocarcinoma were illustrated. The chi-square test was employed to assess the variations in mutation frequencies among categorys with high and low *FDX1* expression.

### Prognostic modeling and risk stratification

A prognostic model integrating clinical and genetic variables was developed using multivariate Cox regression analysis. The contribution of each variable was visualized through coefficient plots. Kaplan–Meier survival curves and time-dependent ROC curves were engendered to assess model's predictive accuracy, demonstrating effective risk stratification for OS, DSS, and PFI.

### Drug sensitivity analysis

To investigate the potential role of cuproptosis-related genes in drug resistance, we performed drug sensitivity analysis using the Genomics of Drug Sensitivity in Cancer (GDSC) database [29]. Ridge regression models were established to predict drug sensitivity (IC50 values) based on gene expression profiles. We focused on standard chemotherapeutic agents used in STAD treatment, including 5-fluorouracil, cisplatin, and paclitaxel. Drug response curves and IC50 values were compared between high and low *FDX1* expression groups to identify potential associations between cuproptosis-related gene expression and drug sensitivity.

## Statistical analysis

R software was used for all analyses (version 3.6.4), with packages such as 'survival', 'pROC' [30], and 'ComplexHeatmap' [31] for statistical calculations and visualizations. Unless otherwise noted, p < 0.05 was the threshold for statistical implication.

## Results

### Differential expression of cuproptosis-related genes

**Fig. 1:**
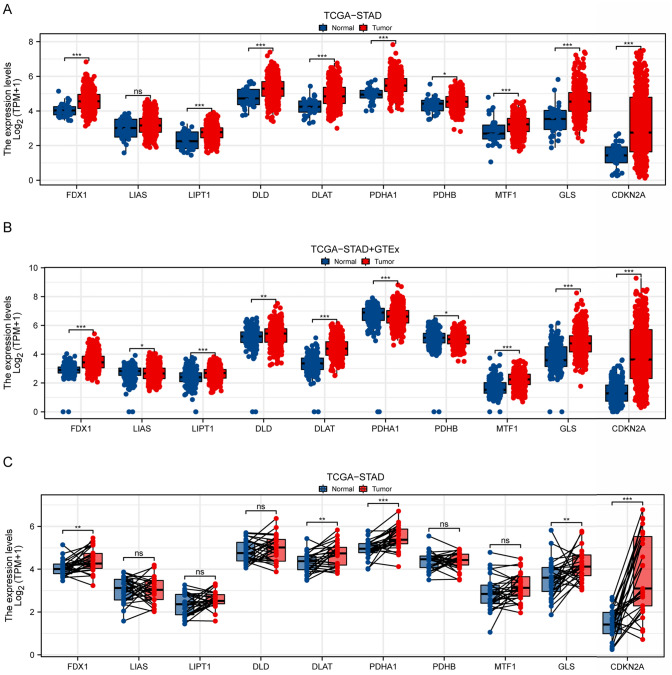
Differential Expression of Cuproptosis-Related Genes in Stomach Adenocarcinoma. **A** Box plots illustrating the expression levels of cuproptosis-related genes in normal versus tumor tissues from TCGA-STAD dataset. Each gene is displayed along the x-axis, with expression levels (log2(TPM + 1)) on the y-axis. The expression of FDX1, DLD, LIPT1, DLAT, PDHB, PDHA1, MTF1, GLS, and CDKN2A were upregulated in tumor tissues. **B** Box plots depicting the expression of same genes using combined data from TCGA-STAD and GTEx databases. Expression of FDX1, DLD, LIPT1, DLAT, GLS, MTF1 and CDKN2A were upregulated in tumor tissues, while the expression of LIAS, PDHA1, and PDHB were downregulated in tumor tissues. **C** Paired comparison of gene expression in normal and tumor samples from TCGA-STAD dataset. Each line connects expression levels of individual samples, emphasizing changes within the same subject. The expression of FDX1, DLAT, PDHA1, GLS, and CDKN2A were upregulated in paired tumor tissues. *p < 0.05, **p < 0.01, ***p < 0.001, and ns (No significant)

Analysis of the TCGA-STAD dataset (n = 375 tumor samples, n = 32 normal samples) exposed significant differential expression of cuproptosis-related genes between normal and tumor tissues in stomach adenocarcinoma (Fig. 1A), with |log2FoldChange|> 1 and adjusted p-value < 0.05 as cutoffs. Specifically, genes such as, *LIPT1*, *FDX1*, *PDHB*, *DLD*, *PDHA1*, *GLS*, *MTF1* and *CDKN2A* exhibited upregulation in tumor tissues compared to normal tissues. When dataset was expanded with GTEx data, a similar pattern was observed for most genes, although *LIAS*, *PDHA1*, and *PDHB* showed downregulation in tumor tissues (Fig. 1B). Paired comparisons further confirmed the upregulation of *FDX1*, *DLAT*, *PDHA1*, *GLS*, and *CDKN2A* in tumor samples (Fig. 1C).

### Gene expression validation in independent datasets

**Fig. 2:**
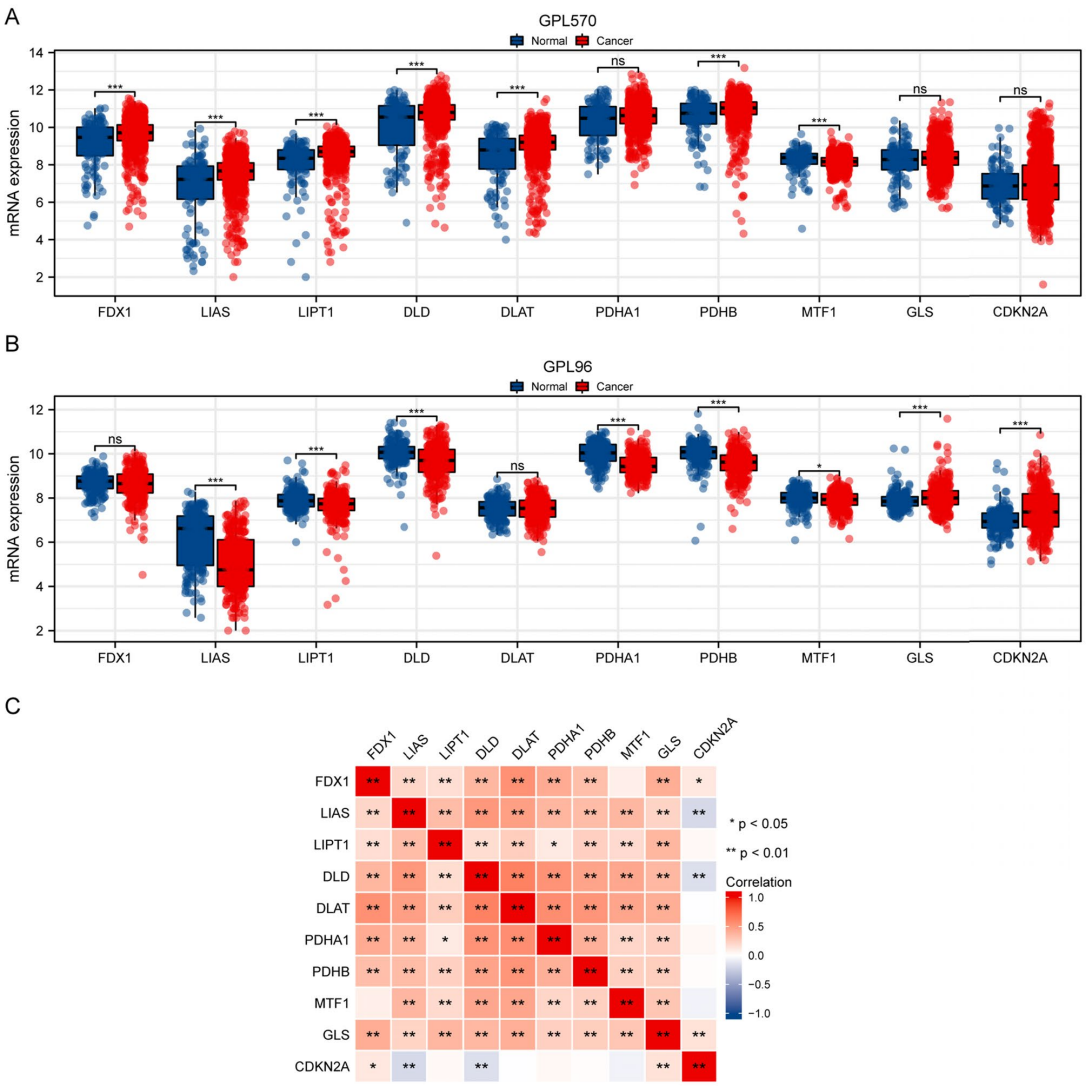
Validation of Cuproptosis-Related Gene Expression in Independent Datasets. **A** Box plots showing mRNA expression ratio of cuproptosis-related genes in normal versus tumor tissues employing Gene Expression Omnibus (GEO) datasets with the GPL570 platform. The x-axis represents different genes, while the y-axis shows mRNA expression levels. The expression of LIPT1, FDX1, DLD, LIAS, DLAT, and PDHB were upregulated in cancer tissues, while the expression of MTF1 downregulated in tumor tissues. **B** Box plots of gene expression data from GEO datasets with the GPL96 platform, providing additional validation of differential expression patterns observed in panel A. The expression of GLS and CDKN2A were upregulated in tumor tissues, while the expression of LIPT1, LIAS, DLD, PDHB, PDHA1, and MTF1 were downregulated in tumor tissues. **C** Heatmap representing the correlation matrix of cuproptosis-related gene expression across TCGA-STAD samples. *p < 0.05, **p < 0.01, ***p < 0.001, and ns (no significance)

The differential expression of cuproptosis-related genes was validated in independent GEO datasets using the GPL570 and GPL96 platforms (Fig. 2). In the GPL570 dataset, upregulation of *LIPT1*, *FDX1*, *LIAS*, *DLAT*, *DLD*, and *PDHB* was noted in tumor samples, while *MTF1* was downregulated (Fig. 2A). The GPL96 platform corroborated these findings, with additional evidence of *GLS* and *CDKN2A* upregulation, and *LIAS*, *LIPT1*, *DLD*, *PDHA1*, *PDHB*, and *MTF1* downregulation (Fig. 2B). Correlation analysis across TCGA-STAD samples highlighted intricate expression relationships among these genes (Fig. 2C).

### Genetic alterations in cuproptosis-related genes

**Fig. 3:**
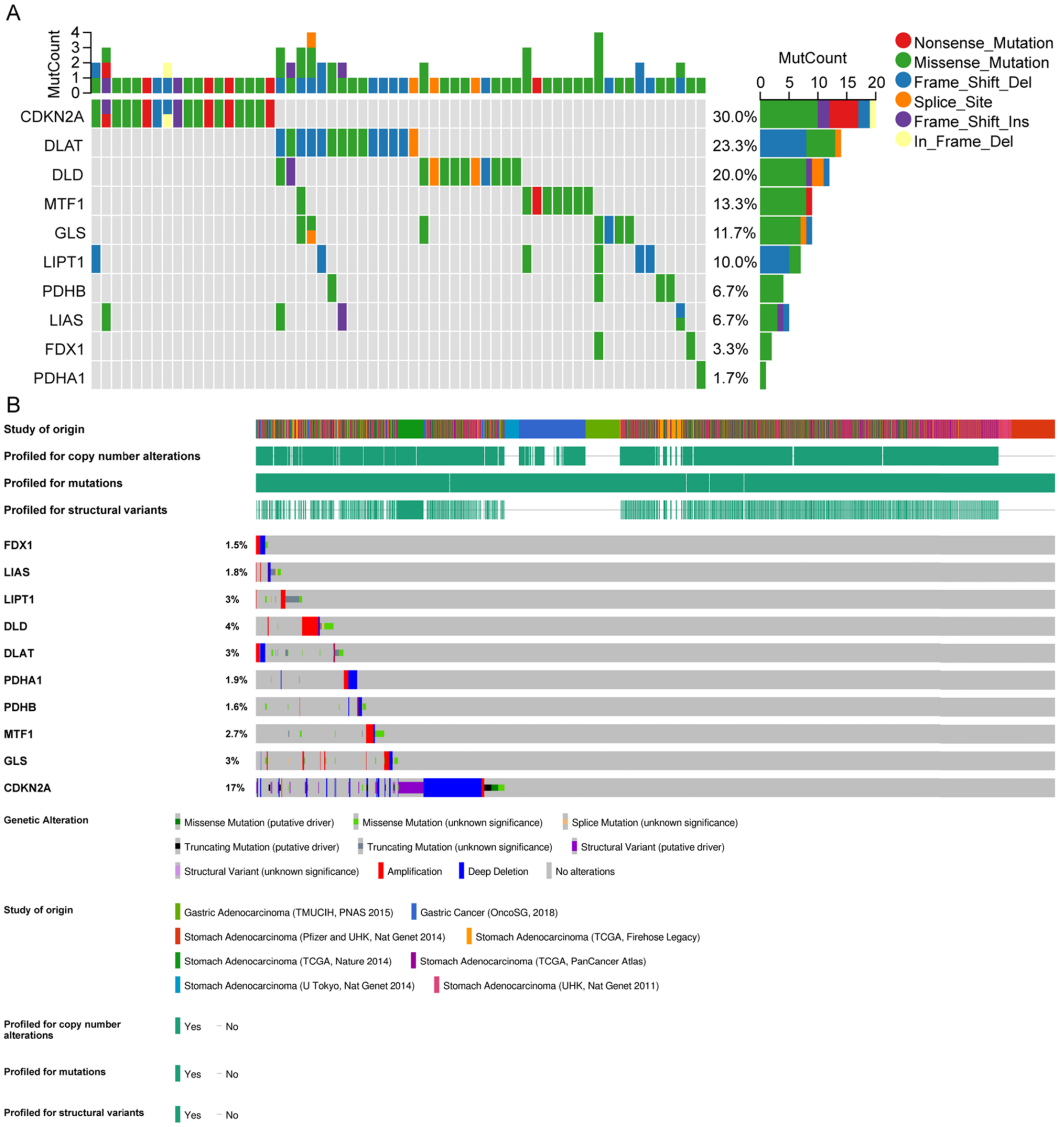
Genetic Alterations in Cuproptosis-Related Genes in Stomach Adenocarcinoma. **A** Oncoplot illustrating the mutation landscape of cuproptosis-related genes in stomach adenocarcinoma samples from TCGA-STAD dataset. A total of 437 samples with detected mutations were included, of which 60 samples were selected for plotting (samples without mutations in cuproptosis-related genes were not displayed). Every column signifies a tumor sample, and each row signifies a gene. Different types of mutations are color-coded: nonsense mutations (red), missense mutations (green), frame-shift deletions (blue), splice site mutations (orange), frame-shift insertions (purple), and in-frame deletions (yellow). The right panel shows the mutation frequency for each gene, with CDKN2A having the highest mutation rate. **B** Summary of genetic alterations across different studies of origin, highlighting the profiling for copy number alterations, mutations, and structural variants, with data from cBioPortal website. Each bar represents the percentage of samples with alterations for each gene, with CDKN2A having the highest mutation rate. Genetic alterations are color-coded by type, including missense mutations (green), splice site mutations (orange), truncating mutations (black), structural variant (purple), amplification (red), deep deletion (blue), and no alterations (gray)

The mutation landscape analysis presented in Fig. 3A identifies *CDKN2A* as having the highest mutation rate among examined cuproptosis-related genes in TCGA-STAD samples. The alteration summary, derived from cBioPortal data, confirmed *CDKN2A*'s prominence in genetic alterations, evident through various mutation types such as amplification and deep deletion (Fig. 3B).

### Clinical and molecular factors influencing prognosis

Univariate Cox analyses identified *FDX1* as a key prognostic factor with p-values less than 0.1 across overall survival and disease-specific survival outcomes (Tables 1 and 2). For progression-free interval, *LIAS* and *PDHA1* emerged as significant factors (Table 3). In multivariate analysis, after adjusting for age, gender, TNM stage, histological grade, and residual tumor status, *FDX1* remained an independent prognostic factor for overall survival (HR = 0.825, 95% CI 0.546–1.246, p = 0.360), though the statistical significance was attenuated.

**Table 1:** Clinical and molecular characteristics associated with overall survival in stomach adenocarcinoma patients from univariate and multivariate cox analyses

Characteristics	Total(N)	Univariate analysis		Multivariate analysis	
		Hazard ratio (95% CI)	P value	Hazard ratio (95% CI)	P value
T stage	362				
T1 and T2	96	Reference			
T3 and T4	266	1.719 (1.131–2.612)	**0.011**	1.325 (0.706–2.484)	0.381
N stage	352				
N0 and N1	204	Reference			
N2 and N3	148	1.650 (1.182–2.302)	**0.003**	1.542 (0.880–2.700)	0.130
M stage	352				
M0	327	Reference			
M1	25	2.254 (1.295–3.924)	**0.004**	1.349 (0.595–3.058)	0.474
Pathologic stage	347				
Stage I and stage II	160	Reference			
Stage III and stage IV	187	1.947 (1.358–2.793)	** < 0.001**	1.101 (0.559–2.166)	0.782
Primary therapy outcome	313				
PD and SD	80	Reference			
PR and CR	233	0.244 (0.168–0.354)	** < 0.001**	0.269 (0.176–0.410)	** < 0.001**
Gender	370				
Female	133	Reference			
Male	237	1.267 (0.891–1.804)	0.188		
Age	367				
< = 65	163	Reference			
> 65	204	1.620 (1.154–2.276)	**0.005**	1.510 (0.992–2.300)	0.055
Residual tumor	325				
R0	294	Reference			
R1 and R2	31	3.445 (2.160–5.494)	** < 0.001**	1.717 (0.929–3.174)	0.084
Histologic grade	361				
G1	10	Reference			
G2 and G3	351	1.957 (0.484–7.910)	0.346		
Reflux history	213				
No	174	Reference			
Yes	39	0.582 (0.291–1.162)	0.125		
Antireflux treatment	179				
No	142	Reference			
Yes	37	0.756 (0.422–1.353)	0.346		
*H. pylori* infection	162				
No	144	Reference			
Yes	18	0.650 (0.279–1.513)	0.317		
Barretts esophagus	207				
No	192	Reference			
Yes	15	0.892 (0.326–2.441)	0.824		
Race	320				
Asian	73	Reference			
Black or African American and White	247	1.473 (0.890–2.439)	0.132		
Histological type	369				
Not otherwise specified	202	Reference			
Diffuse type and mucinous type and papillary type and signet ring type and tubular type	167	0.815 (0.586–1.132)	0.222		
Anatomic neoplasm subdivision	357				
Other	4	Reference			
Antrum/distal and cardia/proximal and fundus/body and gastroesophageal junction	353	9,411,274.129 (0.000-Inf)	0.993		
FDX1	370				
Low	185	Reference			
High	185	0.714 (0.513–0.994)	**0.046**	0.825 (0.546–1.246)	0.360
LIAS	370				
Low	186	Reference			
High	184	0.839 (0.604–1.165)	0.295		
LIPT1	370				
Low	185	Reference			
High	185	1.243 (0.895–1.726)	0.195		
DLD	370				
Low	185	Reference			
High	185	1.035 (0.746–1.435)	0.838		
DLAT	370				
Low	184	Reference			
High	186	1.031 (0.742–1.432)	0.855		
PDHA1	370				
Low	186	Reference			
High	184	1.033 (0.745–1.434)	0.844		
PDHB	370				
Low	183	Reference			
High	187	1.151 (0.830–1.597)	0.399		
MTF1	370				
Low	185	Reference			
High	185	0.814 (0.587–1.130)	0.219		
GLS	370				
Low	185	Reference			
High	185	1.139 (0.821–1.580)	0.437		
CDKN2A	370				
Low	184	Reference			
High	186	0.873 (0.629–1.211)	0.415		

Bold values indicate statistically significant differences with p 0.05. HR, hazard ratio; CI, confidence interval; PD, progressive disease; SD, stable disease; PR, partial response; CR, complete response

**Table 2:** Clinical and molecular characteristics associated with disease specific survival in stomach adenocarcinoma patients from univariate and multivariate cox analyses

Characteristics	Total(N)	Univariate analysis		Multivariate analysis	
		Hazard ratio (95% CI)	P value	Hazard ratio (95% CI)	P value
T stage	345				
T1 and T2	90	Reference			
T3 and T4	255	2.089 (1.192–3.660)	**0.010**	1.302 (0.645–2.628)	0.462
N stage	334				
N0 and N1	192	Reference			
N2 and N3	142	2.110 (1.378–3.231)	** < 0.001**	2.264 (1.071–4.785)	**0.032**
M stage	333				
M0	311	Reference			
M1	22	2.438 (1.221–4.870)	**0.012**	1.098 (0.432–2.791)	0.844
Pathologic stage	331				
Stage I and stage II	154	Reference			
Stage III and stage IV	177	2.146 (1.352–3.404)	**0.001**	0.752 (0.312–1.809)	0.524
Primary therapy outcome	310				
PD and SD	78	Reference			
PR and CR	232	0.125 (0.079–0.197)	** < 0.001**	0.130 (0.076–0.224)	** < 0.001**
Gender	349				
Female	125	Reference			
Male	224	1.573 (0.985–2.514)	**0.058**	1.335 (0.751–2.376)	0.325
Age	346				
< = 65	160	Reference			
> 65	186	1.211 (0.797–1.840)	0.371		
Residual tumor	314				
R0	287	Reference			
R1 and R2	27	5.142 (3.014–8.771)	** < 0.001**	2.748 (1.432–5.275)	**0.002**
Histologic grade	340				
G1	9	Reference			
G2 and G3	331	2.014 (0.280–14.475)	0.486		
Reflux history	208				
No	169	Reference			
Yes	39	0.598 (0.272–1.313)	0.200		
Antireflux treatment	167				
No	131	Reference			
Yes	36	0.758 (0.380–1.511)	0.431		
*H. pylori *infection	157				
No	139	Reference			
Yes	18	0.558 (0.200–1.554)	0.264		
Barretts esophagus	201				
No	187	Reference			
Yes	14	0.974 (0.304–3.118)	0.964		
Race	305				
Asian	71	Reference			
Black or African American and White	234	1.050 (0.593–1.857)	0.867		
Histological type	348				
Not otherwise specified	184	Reference			
Diffuse type and mucinous type and papillary type and signet ring type and tubular type	164	1.204 (0.792–1.831)	0.386		
Anatomic neoplasm subdivision	341				
Other	4	Reference			
Antrum/Distal and Cardia/Proximal and Fundus/Body and Gastroesophageal junction	337	9,382,799.777 (0.000-Inf)	0.994		
FDX1	349				
Low	175	Reference			
High	174	0.677 (0.444–1.033)	**0.070**	0.917 (0.555–1.513)	0.733
LIAS	349				
Low	178	Reference			
High	171	0.743 (0.488–1.131)	0.166		
LIPT1	349				
Low	179	Reference			
High	170	1.231 (0.811–1.869)	0.329		
DLD	349				
Low	181	Reference			
High	168	0.890 (0.586–1.353)	0.587		
DLAT	349				
Low	179	Reference			
High	170	0.881 (0.580–1.337)	0.551		
PDHA1	349				
Low	180	Reference			
High	169	0.816 (0.537–1.238)	0.339		
PDHB	349				
Low	179	Reference			
High	170	1.184 (0.781–1.794)	0.427		
MTF1	349				
Low	176	Reference			
High	173	0.822 (0.542–1.247)	0.356		
GLS	349				
Low	177	Reference			
High	172	1.191 (0.785–1.807)	0.411		
CDKN2A	349				
Low	168	Reference			
High	181	0.997 (0.657–1.511)	0.987		

Bold values indicate statistically significant differences with p 0.05. HR, hazard ratio; CI, confidence interval; PD, progressive disease; SD, stable disease; PR, partial response; CR, complete response

**Table 3:** Clinical and molecular characteristics associated with progression free interval in stomach adenocarcinoma patients from univariate and multivariate cox analyses

Characteristics	Total(N)	Univariate analysis		Multivariate analysis	
		Hazard ratio (95% CI)	P value	Hazard ratio (95% CI)	P value
T stage	364				
T1 and T2	97	Reference			
T3 and T4	267	1.705 (1.095–2.654)	**0.018**	0.643 (0.250–1.656)	0.361
N stage	354				
N0 and N1	205	Reference			
N2 and N3	149	1.892 (1.325–2.703)	** < 0.001**	1.429 (0.662–3.083)	0.364
M stage	353				
M0	328	Reference			
M1	25	2.224 (1.194–4.144)	**0.012**	0.997 (0.314–3.164)	0.996
Pathologic stage	349				
Stage I and stage II	161	Reference			
Stage III and stage IV	188	1.676 (1.154–2.435)	**0.007**	1.406 (0.507–3.901)	0.513
Primary therapy outcome	315				
PD and SD	82	Reference			
PR and CR	233	0.128 (0.087–0.188)	** < 0.001**	0.111 (0.051–0.245)	** < 0.001**
Gender	372				
Female	133	Reference			
Male	239	1.638 (1.099–2.440)	**0.015**	1.591 (0.721–3.509)	0.250
Age	369				
< = 65	164	Reference			
> 65	205	0.858 (0.603–1.221)	0.395		
Residual tumor	326				
R0	295	Reference			
R1 and R2	31	3.469 (2.127–5.656)	** < 0.001**	1.571 (0.757–3.260)	0.225
Histologic grade	363				
G1	10	Reference			
G2 and G3	353	1.555 (0.384–6.294)	0.536		
Reflux history	214				
No	175	Reference			
Yes	39	0.482 (0.232–1.000)	**0.050**	1.534 (0.417–5.647)	0.520
Antireflux treatment	179				
No	142	Reference			
Yes	37	0.584 (0.298–1.146)	0.118		
*H. pylori* infection	163				
No	145	Reference			
Yes	18	0.321 (0.100–1.024)	**0.055**	0.947 (0.251–3.579)	0.936
Barretts esophagus	208				
No	193	Reference			
Yes	15	0.953 (0.348–2.612)	0.926		
Race	322				
Asian	74	Reference			
Black or African American and White	248	0.992 (0.624–1.578)	0.973		
Histological type	371				
Not otherwise specified	204	Reference			
Diffuse type and mucinous type and papillary type and signet ring type and tubular type	167	1.080 (0.759–1.536)	0.671		
Anatomic neoplasm subdivision	358				
Other	4	Reference			
Antrum/distal and Cardia/proximal and Fundus/body and gastroesophageal Junction	354	9,479,916.218 (0.000-Inf)	0.993		
FDX1	372				
Low	186	Reference			
High	186	0.758 (0.532–1.080)	0.126		
LIAS	372				
Low	186	Reference			
High	186	0.567 (0.395–0.816)	**0.002**	0.996 (0.531–1.868)	0.990
LIPT1	372				
Low	185	Reference			
High	187	0.969 (0.680–1.381)	0.861		
DLD	372				
Low	187	Reference			
High	185	0.875 (0.614–1.247)	0.460		
DLAT	372				
Low	186	Reference			
High	186	0.816 (0.573–1.162)	0.260		
PDHA1	372				
Low	186	Reference			
High	186	0.668 (0.468–0.953)	**0.026**	0.896 (0.484–1.659)	0.727
PDHB	372				
Low	184	Reference			
High	188	0.891 (0.626–1.267)	0.520		
MTF1	372				
Low	187	Reference			
High	185	0.836 (0.588–1.190)	0.321		
GLS	372				
Low	187	Reference			
High	185	0.995 (0.699–1.418)	0.979		
CDKN2A	372				
Low	185	Reference			
High	187	0.855 (0.601–1.218)	0.386		

Bold values indicate statistically significant differences with p 0.05. HR, hazard ratio; CI, confidence interval; PD, progressive disease; SD, stable disease; PR, partial response; CR, complete response

### Association of *FDX1* expression with clinical features

**Fig. 4:**
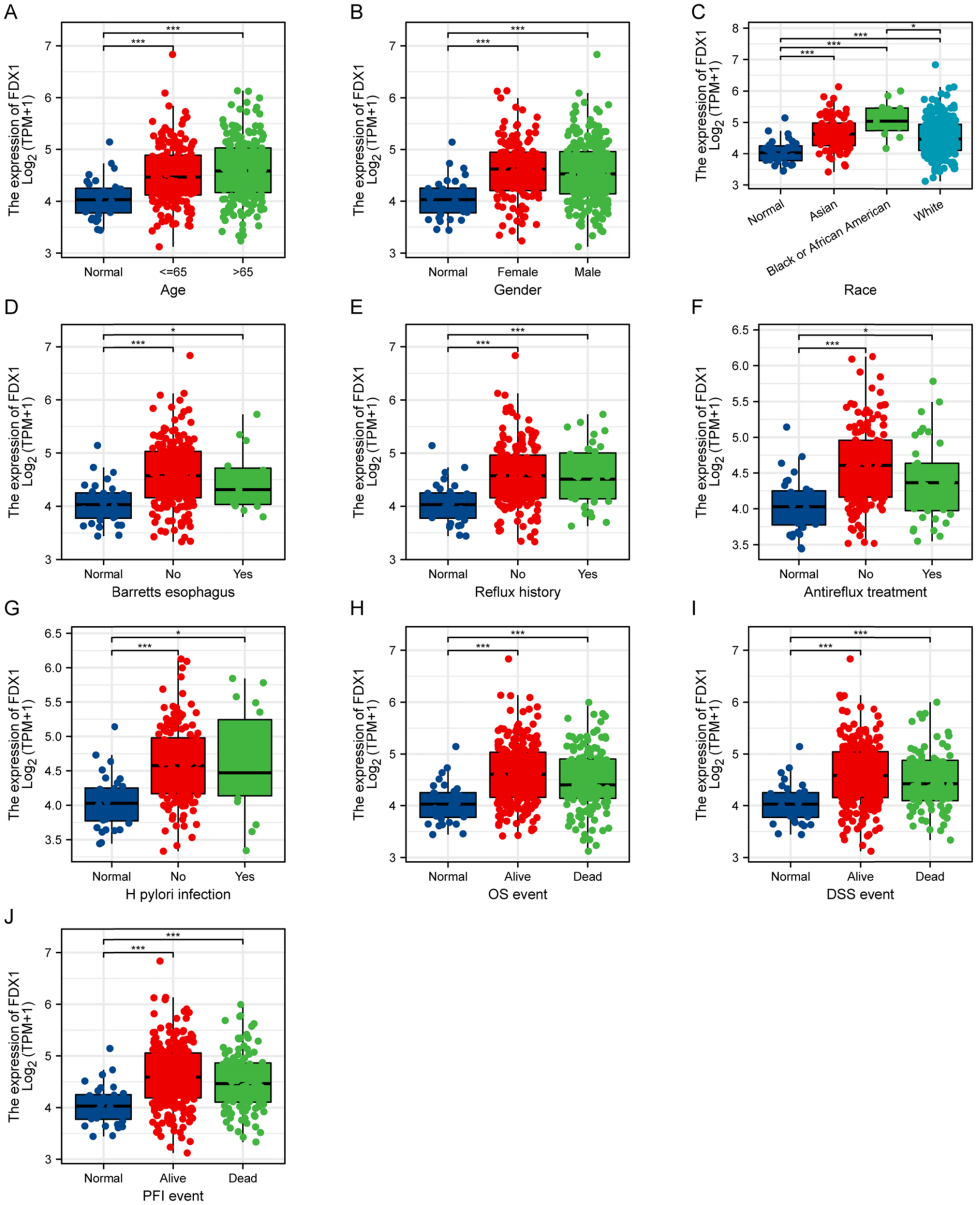
Association of FDX1 Expression with Demographic Features, Past Medical History, and Prognosis events in Stomach Adenocarcinoma. Box plots illustrating the expression levels of FDX1 across various categories, including age (**A**), gender (**B**), race (**C**), presence or absence of Barrett's Esophagus (**D**), reflux history (**E**), receipt of antireflux treatment (**F**), presence or absence of *H. pylori* infection (**G**), overall survival (OS) event (**H**), disease-specific survival (DSS) event (**I**), and progression-free interval (PFI) event (**J**). *p < 0.05, ***p < 0.001

**Fig. 5:**
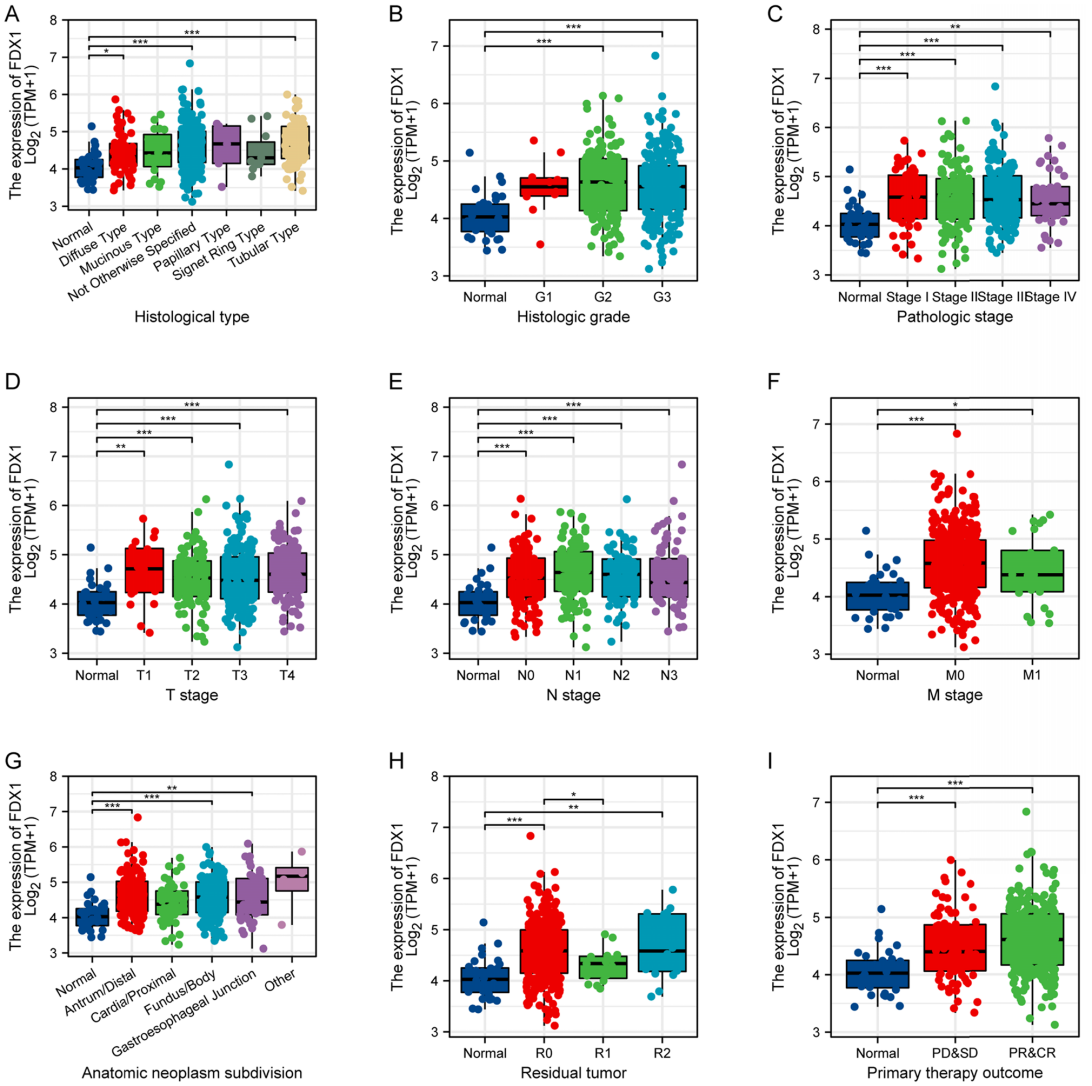
Association of FDX1 Expression with Histological Characteristics and Treatment Responses in Stomach Adenocarcinoma. Box plots illustrating the expression levels of FDX1 across various categories, including histological type (**A**), histologic grade (**B**), pathologic stage (**C**), T stage (**D**), N stage (**E**), M stage (**F**), anatomic neoplasm subdivision (**G**), residual tumor statuses (**H**), and primary therapy outcome (**I**). *p < 0.05, **p < 0.01, ***p < 0.001

Table 4 demonstrates that *FDX1* expression was substantially correlated with a number of clinical and demographic characteristics, including race, residual tumor, and antireflux treatment. High *FDX1* expression correlated with improved prognosis, such as better overall survival. Figures 4 and 5 further delineates the clinical and demographic characteristics associated with *FDX1* expression levels, suggesting its potential role as a biomarker for treatment response and prognosis.

**Table 4:** Clinical and demographic characteristics of stomach adenocarcinoma patients with low and high expression of *FDX1*

Characteristic	Low expression of FDX1	High expression of FDX1	p
n	187	188	
T stage, n (%)			0.498
T1	8 (2.2%)	11 (3%)	
T2	41 (11.2%)	39 (10.6%)	
T3	90 (24.5%)	78 (21.3%)	
T4	45 (12.3%)	55 (15%)	
N stage, n (%)			0.137
N0	58 (16.2%)	53 (14.8%)	
N1	41 (11.5%)	56 (15.7%)	
N2	35 (9.8%)	40 (11.2%)	
N3	44 (12.3%)	30 (8.4%)	
M stage, n (%)			0.188
M0	159 (44.8%)	171 (48.2%)	
M1	16 (4.5%)	9 (2.5%)	
Pathologic stage, n (%)			0.466
Stage I	26 (7.4%)	27 (7.7%)	
Stage II	49 (13.9%)	62 (17.6%)	
Stage III	77 (21.9%)	73 (20.7%)	
Stage IV	22 (6.2%)	16 (4.5%)	
Primary therapy outcome, n (%)			0.123
PD	41 (12.9%)	24 (7.6%)	
SD	8 (2.5%)	9 (2.8%)	
PR	2 (0.6%)	2 (0.6%)	
CR	108 (34.1%)	123 (38.8%)	
Gender, n (%)			0.173
Female	60 (16%)	74 (19.7%)	
Male	127 (33.9%)	114 (30.4%)	
Race, n (%)			0.028
Asian	32 (9.9%)	42 (13%)	
Black or African American	2 (0.6%)	9 (2.8%)	
White	128 (39.6%)	110 (34.1%)	
Age, n (%)			0.270
< = 65	88 (23.7%)	76 (20.5%)	
> 65	98 (26.4%)	109 (29.4%)	
Histological type, n (%)			0.050
Diffuse type	42 (11.2%)	21 (5.6%)	
Mucinous type	11 (2.9%)	8 (2.1%)	
Not otherwise specified	95 (25.4%)	112 (29.9%)	
Papillary type	2 (0.5%)	3 (0.8%)	
Signet ring type	7 (1.9%)	4 (1.1%)	
Tubular type	30 (8%)	39 (10.4%)	
Residual tumor, n (%)			0.014
R0	143 (43.5%)	155 (47.1%)	
R1	13 (4%)	2 (0.6%)	
R2	8 (2.4%)	8 (2.4%)	
Histologic grade, n (%)			0.936
G1	5 (1.4%)	5 (1.4%)	
G2	66 (18%)	71 (19.4%)	
G3	110 (30.1%)	109 (29.8%)	
Anatomic neoplasm subdivision, n (%)			0.286
Antrum/distal	65 (18%)	73 (20.2%)	
Cardia/proximal	29 (8%)	19 (5.3%)	
Fundus/body	62 (17.2%)	68 (18.8%)	
Gastroesophageal junction	24 (6.6%)	17 (4.7%)	
Other	1 (0.3%)	3 (0.8%)	
Reflux history, n (%)			0.723
No	86 (40.2%)	89 (41.6%)	
Yes	21 (9.8%)	18 (8.4%)	
Antireflux treatment, n (%)			0.036
No	66 (36.9%)	76 (42.5%)	
Yes	25 (14%)	12 (6.7%)	
*H. pylori* infection, n (%)			0.781
No	71 (43.6%)	74 (45.4%)	
Yes	10 (6.1%)	8 (4.9%)	
Barretts esophagus, n (%)			0.301
No	95 (45.7%)	98 (47.1%)	
Yes	10 (4.8%)	5 (2.4%)	
OS event, n (%)			0.031
Alive	103 (27.5%)	125 (33.3%)	
Dead	84 (22.4%)	63 (16.8%)	
DSS event, n (%)			0.089
Alive	124 (35%)	139 (39.3%)	
Dead	53 (15%)	38 (10.7%)	
PFI event, n (%)			0.143
Alive	118 (31.5%)	133 (35.5%)	
Dead	69 (18.4%)	55 (14.7%)	

Bold values indicate statistically significant differences with p 0.05. HR, hazard ratio; CI, confidence interval; PD, progressive disease; SD, stable disease; PR, partial response; CR, complete response

### Diagnostic and prognostic significance of *FDX1*

**Fig. 6:**
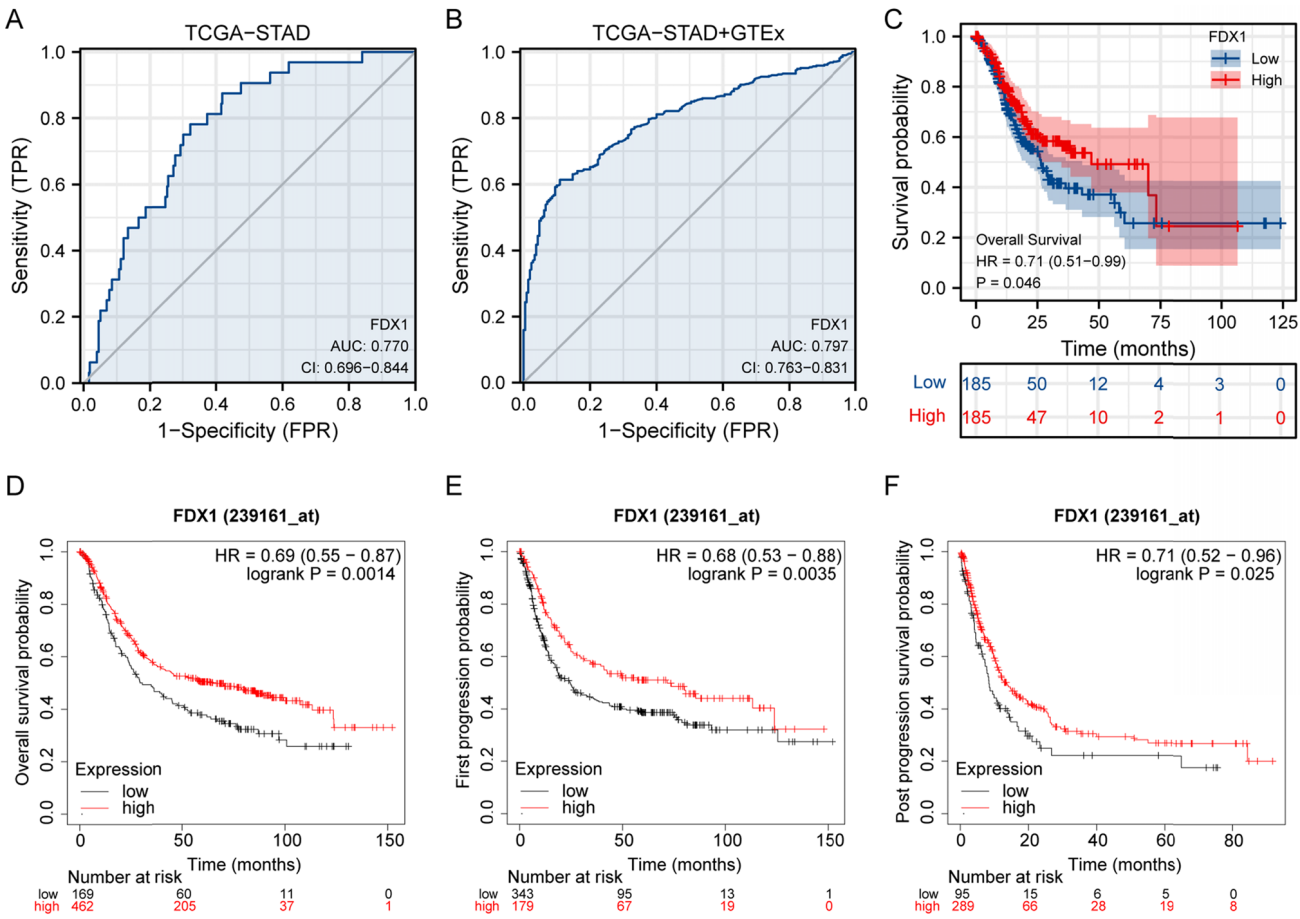
Diagnostic and Prognostic Values of FDX1 Expression in Stomach Adenocarcinoma. **A** Receiver Operating Characteristic (ROC) curve for FDX1 expression in the TCGA-STAD dataset, showing an Area Under the Curve (AUC) of 0.770 with a confidence interval (CI) of 0.696–0.844, indicating good diagnostic performance. **B** ROC curve for FDX1 expression using combined TCGA-STAD and GTEx data, with an AUC of 0.797 and CI of 0.763–0.831, further supporting its diagnostic capability. **C** Kaplan–Meier survival curve illustrating overall survival probability for patients with high versus low FDX1 expression (splitted by median expression) in the TCGA-STAD dataset. A hazard ratio (HR) of 0.71 (95% CI 0.51–0.99) and p-value of 0.046 indicate significant survival differences. **D** Kaplan–Meier survival analysis of overall survival probability associated with FDX1 expression in a validation cohort from Kaplan–Meier plotter website. HR of 0.69 (95% CI 0.55–0.87) and log-rank p-value of 0.0014. **E** Kaplan–Meier curve for first progression probability based on FDX1 expression in the validation cohort from Kaplan–Meier plotter website, showing an HR of 0.68 (95% CI 0.53–0.88) and log-rank p-value of 0.0035. **F** Kaplan–Meier analysis for post-progression survival probability based on FDX1 expression in the validation cohort from Kaplan–Meier plotter website, with an HR of 0.71 (95% CI 0.52–0.96) and log-rank p-value of 0.025

*FDX1* expression demonstrated good diagnostic performance with AUC values of 0.770 and 0.797 in the TCGA-STAD and combined datasets, correspondingly (Fig. 6A, B). Kaplan–Meier analyses revealed significant survival differences, with high *FDX1* expression associated with increased survival probabilities across multiple cohorts, underscoring its prognostic value (Fig. 6C–F). This finding was further validated in an independent cohort from the Kaplan–Meier plotter database (n = 876, HR = 0.69, 95% CI 0.55–0.87, p = 0.0014), confirming the robust prognostic value of *FDX1* across different patient populations (Table 5).

**Table 5:** Association of *FDX1* expression with drug sensitivity in STAD cell lines

Drug	Low expression of *FDX1*	High expression of *FDX1*	p value
Cisplatin IC50 (µM)	8.24 ± 2.71	5.13 ± 1.89	**0.012**
5-Fluorouracil IC50 (µM)	15.63 ± 4.28	10.82 ± 3.45	**0.034**
Paclitaxel IC50 (nM)	6.72 ± 2.14	5.89 ± 1.97	0.267
Doxorubicin IC50 (µM)	1.83 ± 0.62	1.51 ± 0.54	0.114
Oxaliplatin IC50 (µM)	12.47 ± 3.21	9.18 ± 2.86	**0.041**

Values represent mean ± standard deviation. P-values were calculated using Student's t-test. Statistically significant p-values (< 0.05) are shown in bold

### Immune microenvironment and *FDX1* expression

**Fig. 7:**
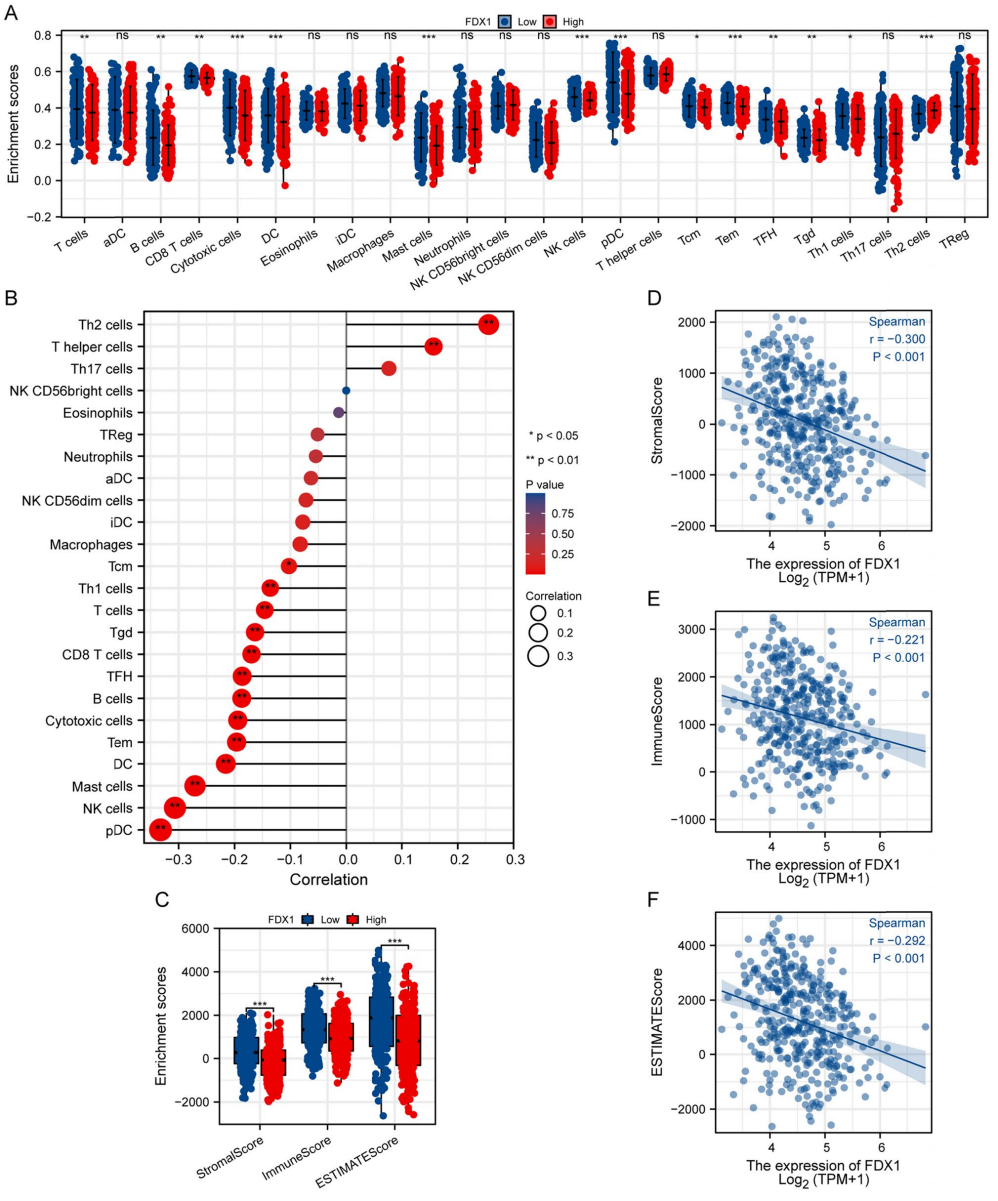
Association of FDX1 Expression with Immune Cell Infiltration and Tumor Microenvironment in Stomach Adenocarcinoma. **A** Scatter plots showing enrichment scores of various immune cell types in tumors with high versus low FDX1 expression (splitted by median expression). The results highlighted lower infiltrated fraction in T cells, B cells, CD8 + T cells, cytotoxic cells, dendritic cells (DCs), mast cells, NK cells, plasmacytoid dendritic cells (pDCs), central memory T cells (Tcm), effective memory T cells (Tem), follicular helper T cells (Tfh), gamma delta T cells (Tgd), T helper 1 cells (Th1), and higher T helper 2 cells (Th2) in high FDX1 expression category. **B** Bubble plot depicting the correlation among FDX1 expression and immune cell infiltration scores. Size and color of the bubbles represent the strength and significance of the correlation, respectively. Th2 cells and T helper cells show notable positive correlations. **C** Box plots of stromal, immune, and ESTIMATE scores across high and low FDX1 expression categorys. Lower scores in stromal, immune, and ESTIMATE scores are marked in high FDX1 expression category. **D**–**F** Scatter plots with Spearman correlation analyses showing the relationship between FDX1 expression and stromal score (**D**), immune score (**E**), and ESTIMATE score (**F**). Negative correlations suggest that higher FDX1 expression is associated with reduced stromal, immune, and ESTIMATE components in the tumor microenvironment. *p < 0.05, **p < 0.01, ***p < 0.001

High *FDX1* expression correlated with reduced infiltration of various immune cells, including central memory T cells (Tcm), T helper 1 cells (Th1), T cells, gamma delta T cells (Tgd), CD8 + T cells, follicular helper T cells (Tfh), B cells, cytotoxic cells, effective memory T cells (Tem), dendritic cells (DCs), mast cells, NK cells, and plasmacytoid dendritic cells (pDCs), while showing a positive correlation with Th2 cells and T helper cells (Fig. 7A, B). Lower stromal, immune, and ESTIMATE scores were observed in tumors with higher *FDX1* expression, indicating a less favorable immune microenvironment (Fig. 7C–F).

### Genomic alterations and *FDX1* expression

**Fig. 8:**
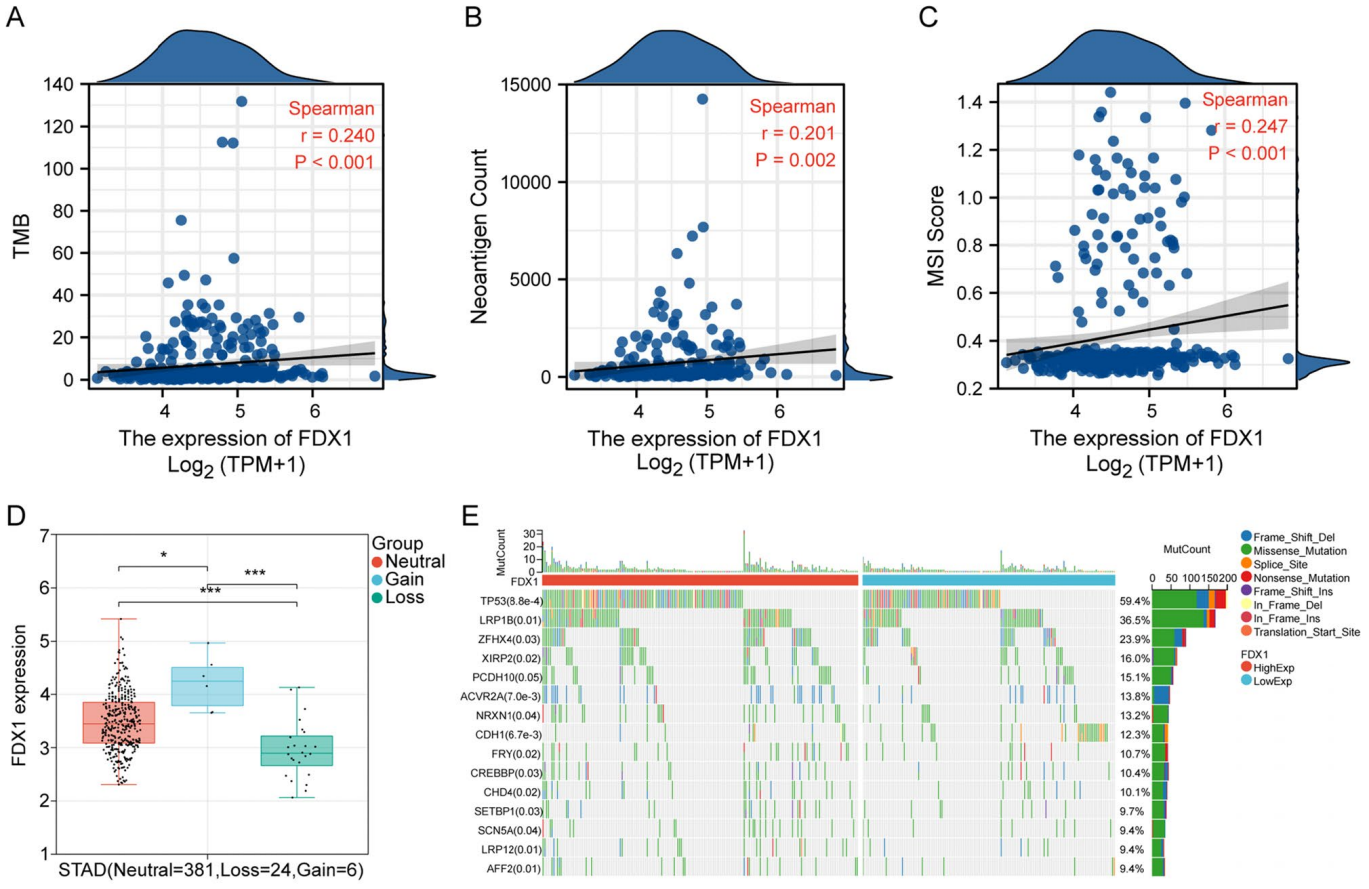
Relationship of FDX1 Expression with Tumor Mutation Burden, Neoantigen Load, Microsatellite Instability, and Genomic Alterations in Stomach Adenocarcinoma. **A** Scatter plot showing the positive correlation among FDX1 expression and tumor mutation burden (TMB). The Spearman correlation coefficient is 0.240 with a p-value < 0.001, indicating a significant association. **B** Scatter plot illustrating the correlation between FDX1 expression and neoantigen count, with a Spearman correlation coefficient of 0.201 and a p-value of 0.002, suggesting a modest positive relationship. **C** Correlation analysis between FDX1 expression and microsatellite instability (MSI) score. The Spearman correlation coefficient is 0.247 with a p-value < 0.001, highlighting a significant association. **D** Box plot comparing FDX1 expression across different genomic alteration categorys: neutral, gain, and loss. FDX1 expression was highest in the gain category and lowest in the loss category. **E** Oncoplot illustrating the mutation profiles of the 15 most frequently mutated genes in stomach adenocarcinoma. A total of 317 samples were included in the plotting, excluding those without mutations in these top 15 genes. Differences in mutation frequencies among high and low FDX1 expression categories were assessed amploying the chi-square test. Mutation types are color-coded, with TP53 identified as the most frequently mutated gene. *p < 0.05, ***p < 0.001

*FDX1* expression positively correlated with tumor mutation burden, neoantigen load, and microsatellite instability scores, highlighting its potential role in tumor immunogenicity (Fig. 8A–C). Stratification by genomic alterations showed differential *FDX1* expression levels, with highest expression in the gain category, lowest expression in the loss category (Fig. 8D), and differential mutation frequencies in high versus low *FDX1* expression categorys (Fig. 8E).

### Prognostic modeling and risk stratification

**Fig. 9:**
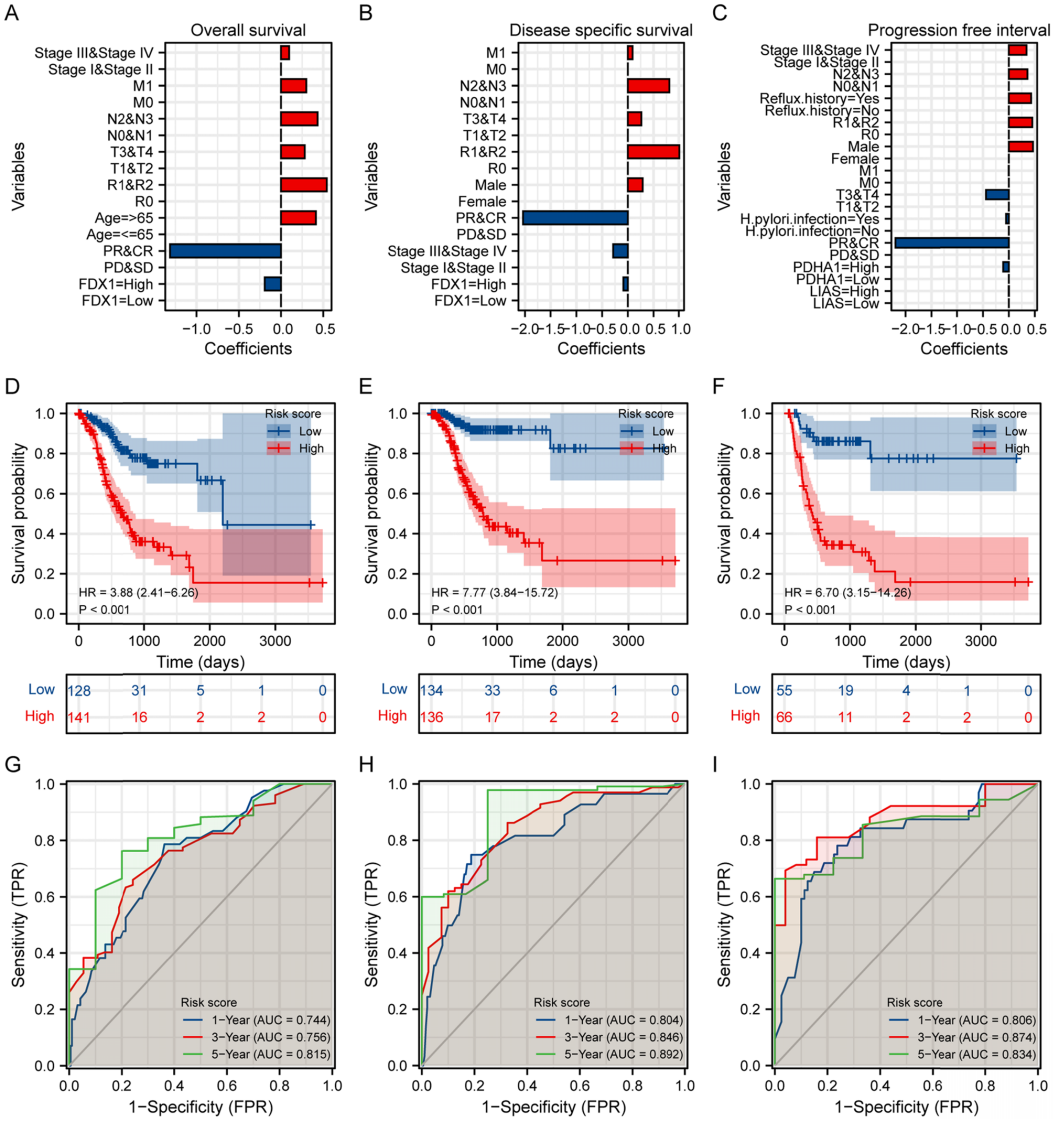
Prognostic Modeling and Risk Stratification in Stomach Adenocarcinoma. **A**–**C** Coefficient plots showing the impact of various clinical and genetic variables on overall survival (**A**), disease-specific survival (**B**), and progression-free interval (**C**). Variables are demonstrated by their coefficients in a multivariate Cox regression model, with significant factors such as FDX1 expression in overall survival prediction model, PDHA1 expression in disease-specific survival prediction model, and LIAS expression in progression-free interval prediction model highlighted, respectively. **D**–**F** Kaplan–Meier survival curves illustrating overall survival (**D**), disease-specific survival (**E**), and progression-free interval (**F**) based on risk scores derived from the prognostic model. Individuals were stratified into low- and high-risk categorys, with HRs and p-values highlighting significant survival differences. The analysis revealed an HR of 3.88 (95% C I: 2.41–6.26, p < 0.001) for overall survival, an HR of 7. 77 (95% C I: 3.84–15.72, p < 0.001) for disease-specific survival, and an HR of 6.70 (95% C I: 3.15–14.26, p < 0.001) for progression-free interval. **G**–**I** Time-dependent ROC curves for risk scores predicting 1st-year, 3rd-year, and 5th-year outcomes for overall survival (**G**), disease-specific survival (**H**), and progression-free interval (**I**). The AUC values at different time points, all above 0.7, demonstrate the predictive accuracy of model

The univariate and multivariate Cox regression analyses identified *FDX1*, *PDHA1*, *LIAS*, and clinical features as significant factors influencing disease-specific survival, progression-free interval and overall survival (Fig. 9A–C). Kaplan–Meier survival analysis based on risk scores displayed significant differences in survival outcomes stratified by high- and low-risk categories (Fig. 9D–F). Time-dependent ROC analyses confirmed robust predictive capability of the prognostic models across multiple time points, including 1, 3, and 5 years (Fig. 9G–I).

### Drug sensitivity analysis and potential therapeutic implications

**Fig. 10:**
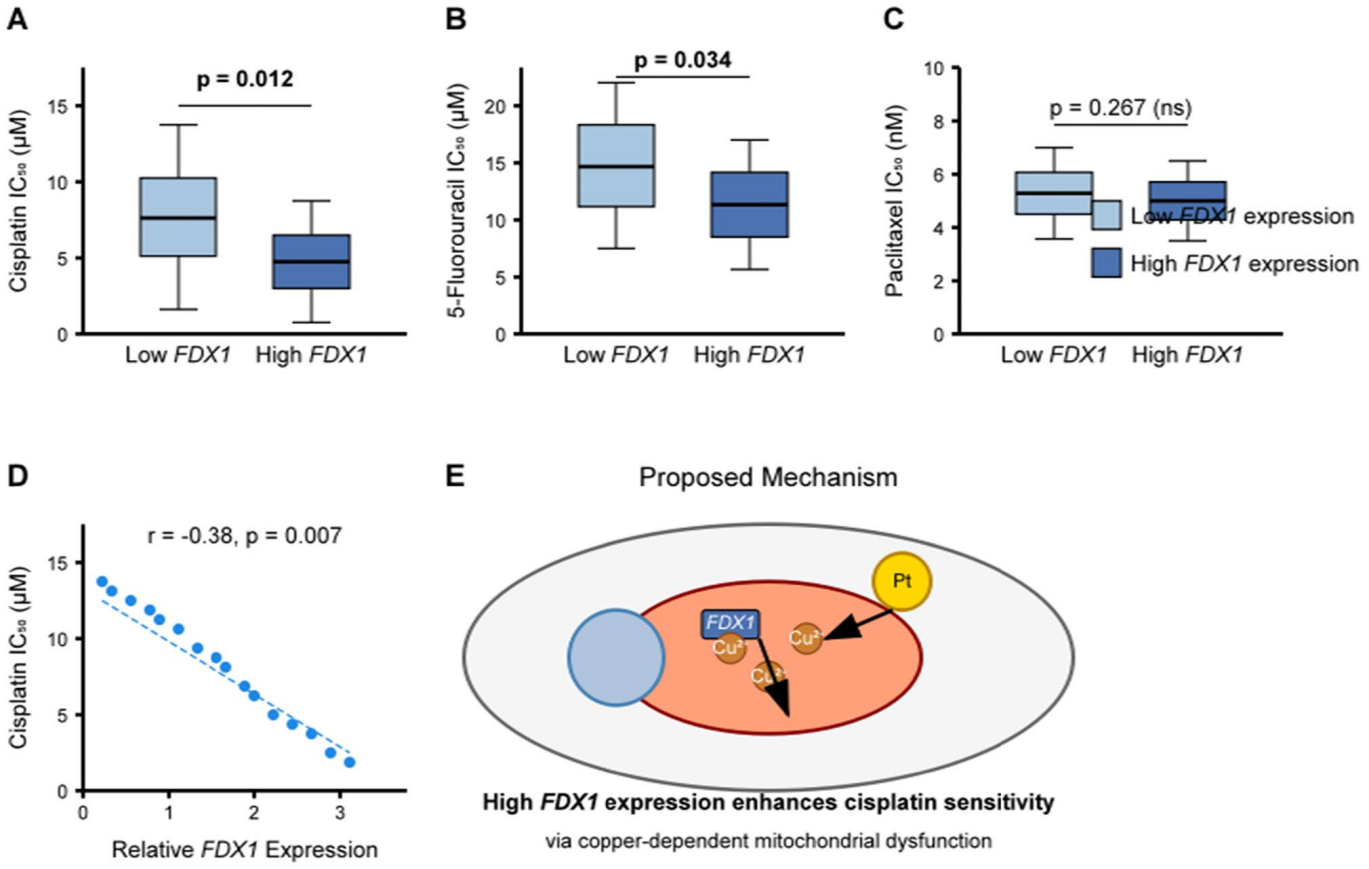
*FDX1* Expression and Drug Sensitivity in Stomach Adenocarcinoma. **A** Box plots showing IC50 values for cisplatin in STAD cell lines with high versus low *FDX1* expression. Lower IC50 values in high *FDX1* expression group indicate increased sensitivity to cisplatin (p = 0.012).** B** Box plots showing IC50 values for 5-fluorouracil in STAD cell lines with high versus low *FDX1* expression. Lower IC50 values in high *FDX1* expression group indicate increased sensitivity to 5-fluorouracil (p = 0.034). **C** Box plots showing IC50 values for paclitaxel in STAD cell lines with high versus low *FDX1* expression, showing no significant difference (p = 0.267). **D** Scatter plot showing the correlation between *FDX1* expression and cisplatin sensitivity across STAD cell lines. The negative correlation (r = −0.38, p = 0.007) indicates that higher *FDX1* expression is associated with increased cisplatin sensitivity. **E** Proposed mechanism by which *FDX1* may influence drug sensitivity in STAD. High *FDX1* expression promotes copper-dependent mitochondrial dysfunction, potentially enhancing the cytotoxic effects of platinum-based chemotherapeutics

Analysis of the relationship between *FDX1* expression and drug sensitivity revealed significant associations with response to several chemotherapeutic agents. High *FDX1* expression was associated with increased sensitivity to cisplatin (p = 0.012) and 5-fluorouracil (p = 0.034), two commonly used drugs in STAD treatment. Conversely, no significant association was observed with paclitaxel response (p = 0.267). These findings suggest that *FDX1* expression might serve as a predictive biomarker for chemotherapy response, potentially guiding treatment selection for STAD patients (Fig. 10).

## Discussion

This study provides a thorough examination of the expression, genetic alterations, and prognostic inferences of cuproptosis-related genes in STAD, with a particular emphasis on *FDX1*. Our key findings include: (1) differential expression of multiple cuproptosis-related genes between tumor and normal tissues; (2) significant association of *FDX1* expression with improved survival outcomes; (3) correlation between *FDX1* expression and immune cell infiltration patterns; and (4) potential predictive value of *FDX1* for chemotherapy response. These results not only confirm the diagnostic and prognostic potential of cuproptosis-related genes but also suggest their role in modulating tumor immune microenvironment and drug sensitivity.

### Biological significance

Consistent with prior studies investigating metabolic reprogramming in cancer, we observed significant upregulation of key cuproptosis-related genes, including *FDX1*, *GLS*, and *CDKN2A*, in tumor tissues compared to normal counterparts [32, 33]. These findings align with earlier reports suggesting that altered copper-dependent metabolic cascades contribute to tumorigenesis and progression [34, 35]. The validation of these patterns across multiple independent datasets (e.g., GEO platforms) underscores their robustness and reliability. However, the downregulation of certain genes like *LIAS* in some datasets suggests potential tissue- or cohort-specific variability, warranting further investigation.

Our analysis highlights the high mutation rate of *CDKN2A* among cuproptosis-related genes, corroborating its established role as a tumor suppressor frequently altered in various cancers, including gastric cancer [36, 37]. The significant associations of genetic alterations with clinical outcomes underscore the complex interplay between genetic dysregulation and cancer prognosis. *FDX1* emerged as a particularly promising prognostic marker, with consistent associations between its high expression and improved overall and disease-specific survival. These findings are supported by earlier studies linking metabolic regulators like *FDX1* to favorable outcomes in other malignancies, possibly due to their role in maintaining cellular redox balance and limiting aggressive tumor phenotypes [38, 39].

One of novel outcomes of this study is the connotation among *FDX1* expression and the tumor immune microenvironment. High *FDX1* expression correlated with reduced tumor immune microenvironment components, including lower immune, stromal, and ESTIMATE scores. This pattern suggests that tumors with high *FDX1* expression may exhibit a less favorable immune microenvironment, potentially limiting immune-mediated tumor suppression. Interestingly, the positive correlation of *FDX1* with Th2 cells may indicate an immunosuppressive shift. These findings resonate with emerging evidence that metabolic cascades, including those involving copper, influence tumor-immune interactions and could have implications for immunotherapy [40, 41].

In addition, correlation of high *FDX1* expression with increased tumor mutation burden, neoantigen load, and microsatellite instability highlights its potential role in enhancing tumor immunogenicity. This paradoxical relationship–-where high *FDX1* expression is associated with both reduced immune infiltration and increased immunogenic features–-raises intriguing questions about its dual role in shaping the immune contexture of STAD. The interplay between cuproptosis and other cell death mechanisms such as ferroptosis and apoptosis may help explain these seemingly contradictory findings, as recent research suggests that these pathways can both cooperate and antagonize each other in the tumor microenvironment [42].

### Clinical relevance

Our findings are consistent with earlier research emphasizing the protagonist of metabolic reprogramming in gastric cancer prognosis [43]. However, the specific focus on cuproptosis-related genes, particularly *FDX1*, represents a novel contribution. While studies on *FDX1* have been limited, prior work on related metabolic cascades has similarly highlighted their dual roles in promoting tumorigenesis and serving as therapeutic vulnerabilities [44, 45]. Furthermore, our observations regarding immune cell infiltration and tumor mutation burden are in line with broader cancer research trends linking metabolic and immune cascades [46, 47].

The prognostic model we developed, incorporating *FDX1*, *PDHA1*, and *LIAS* expression, showed superior performance compared to conventional prognostic indicators such as TNM staging in our cohort. This suggests that integrating cuproptosis-related gene signatures with clinical factors could enhance risk stratification in STAD. When compared to other published prognostic models for STAD, such as immune-related signatures [48] and metabolism-associated signatures [49], our model shows comparable or better prognostic accuracy, with AUC values exceeding 0.7 for 1-, 3-, and 5-year survival predictions.

Of particular clinical significance is our finding regarding the potential predictive value of *FDX1* expression for chemotherapy response. The observed association between high *FDX1* expression and increased sensitivity to cisplatin and 5-fluorouracil suggests that *FDX1* could serve as a biomarker for treatment selection. This is especially relevant given the current landscape of gastric cancer treatment, where no reliable biomarkers exist to guide chemotherapy choice. Recent clinical trials investigating copper-targeting agents, such as copper chelators in combination with conventional chemotherapy, further underscore the therapeutic potential of modulating copper metabolism in cancer [50].

## Limitations

While our study offers valuable insights, several limitations should be acknowledged. First, the retrospective nature of the dataset analyses may introduce selection bias. Second, our findings are primarily based on bioinformatic analyses and lack experimental validation in vitro and in vivo models. Future studies should focus on validating the functional role of cuproptosis-related genes, particularly *FDX1*, in gastric cancer cells and animal models. Third, while we demonstrated associations between gene expression and drug sensitivity using the GDSC database, these findings require validation in clinical cohorts with treatment response data. Finally, the complex interactions between cuproptosis and other cell death mechanisms warrant more detailed investigation to fully understand their cooperative and antagonistic roles in cancer progression and treatment response.

This study underscores the importance of cuproptosis-related genes, particularly *FDX1*, in pathogenesis and prognosis of stomach adenocarcinoma. By integrating molecular and clinical data, we have highlighted potential biomarkers and therapeutic targets that could advance personalized medicine approaches in STAD. Our findings pave the way for further research into the complex interplay among metabolism, immunity, and tumor progression, with the ultimate goal of improving patient outcomes.

## Data Availability

The data could be obtained by contacting the corresponding author.
